# Gut Signals and Gut Feelings: Science at the Interface of Data and Beliefs

**DOI:** 10.3389/fnbeh.2022.929332

**Published:** 2022-07-05

**Authors:** Peter Holzer

**Affiliations:** Division of Pharmacology, Otto Loewi Research Centre, Medical University of Graz, Graz, Austria

**Keywords:** brain-gut axis, gut-brain axis, interoception, emotion, affect, cognition, beliefs

## Introduction

The discovery that signals from the gut reaching the brain can have an impact on affect, emotion and cognition including beliefs and decision-making has been met with considerable public attention. As discussed here, widespread interest in a research field that touches one's personal health also raises expectations and beliefs that are not thoroughly backed by validated scientific evidence. The term “gut feelings,” derived from a lay interpretation of the impact of gut signals on mental activity, is a popular but scientifically ill-defined term that may even lead science astray from its principles of investigation. In these interdependencies, the relation between gut signals and “gut feelings” is a worthwhile theme for analysis in credition research. As outlined in the overview of the Special Topic that this article is part of, credition research is an interdisciplinary approach to understand the nature of beliefs and believing. Based on distinct neuronal processes in the brain, credition refers to the integrative processing of information, its valuation in terms of personal meaning and its impact on a person's behavioral decisions (Seitz et al., 2018; Seitz, 2022).

## Bidirectional Gut-Brain Communication

**Figure 1:**
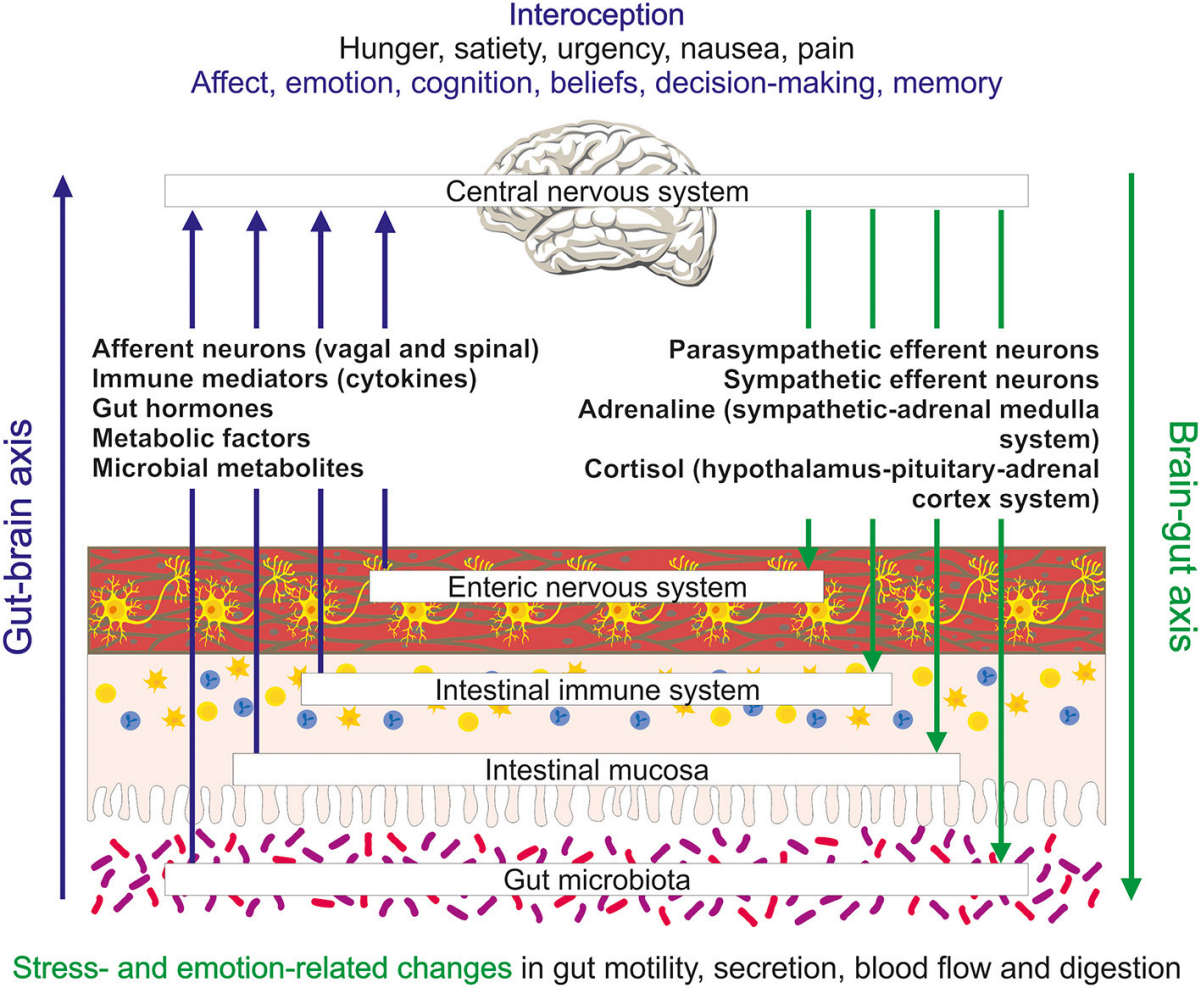
Schematic illustration of the bidirectional exchange of information between the gut and brain and the interoceptive processing of this information in the brain. Several messenger systems including extrinsic afferent neurons, immune mediators, gut hormones, metabolic factors and microbial metabolites carry information from the gut to the brain. They elicit conscious sensations (e.g., hunger, satiety, urgency, nausea, and pain) and influence processes relevant to affect, emotion and cognition. Regulatory outputs from the brain to the gut include emotion- and stress-related changes in motility, secretion, blood flow and digestion.

Psychosomatic medicine has gathered ample evidence that gastrointestinal function can be altered by emotions and stress. For instance, 75 years ago Almy and Tulin (1947) published a study in which they performed sigmoidoscopies in volunteer medical students. When during the examination they told the students that they had discovered a carcinoma, they instantly observed strong muscle contractions and an increase in blood flow in the rectum. Once they explained the hoax, the uproar in the rectum subsided rapidly. Although this kind of study would no longer receive ethical approval (Shea-Donohue et al., 2005), it shows that emotional stress can have an immediate impact on the gut. Since then, many studies have confirmed (for reviews see Mayer, 2000; Taché et al., 2001), in a more humane way, that acute physical and emotional stress can affect the digestive tract in a regionally distinct manner, retarding gastric emptying (“being heavy on the stomach”) but hastening colonic propulsion (“soiling one's pants”). The changes in gut function accompanying long-term stress, however, may substantially differ from those in acute settings (McEwen, 2007). The communication from the brain to the gut, often referred to as “brain-gut axis,” is transmitted by several pathways (Figure 1) including the efferent autonomic nervous system with its sympathetic and parasympathetic divisions and neuroendocrine factors of the sympathetic-adrenal medulla and hypothalamus-pituitary-adrenal cortex systems (Holzer et al., 2015).

Communication between brain and gut is in fact a bidirectional process that, besides efferent connections, also involves afferent pathways that carry information from the gastrointestinal tract to the central nervous system (CNS) (Figure 1). Extrinsic sensory neurons of the vagal and spinal nerves transmitting mechanical and chemical stimuli are one component of this “gut-brain axis.” Endocrine chemical messages carried by gut hormones released from enteroendocrine cells in the gut mucosa, mediators (cytokines) of the gastrointestinal immune system, metabolic factors related to nutrient absorption and digestion, and messengers generated by the gut microbiota (Figure 1) constitute other important components of the gut-brain communication network. The enteric nervous system intrinsic to the gut (Perez-Burgos et al., 2014), enteroendocrine cells (Kaelberer et al., 2020) as well as immune and microbial messengers also use, in part, extrinsic sensory neurons to signal to the brain (Holzer et al., 2015).

This complex afferent communication system provides the brain with integrated information on gut function. In this task, the gut-brain axis contributes to interoception, a process that enables the brain to “know” the internal state of the body (Craig, 2002) and align its mental activity and homeostatic body control (Chen et al., 2021). Functional imaging studies have shown that information coming from the gut reaches brain regions relevant to emotion, affect and cognition. A good deal of what we now know about the bidirectional information exchange between gut and brain has been disclosed by research efforts to understand irritable bowel syndrome (IBS). Characterized by recurrent abdominal pain associated with alterations in bowel habits, IBS is frequently comorbid with anxiety disorders and depression and now widely considered a disorder of gut-brain-gut communication (Black et al., 2020a; Mayer et al., 2022).

## Interoceptive Gut-Brain Communication: Impact on Mental Processes

The complex gut-brain-gut communication network contributes to interoception (Mayer, 2011; Holzer, 2017), a process that integrates information from all internal organs to impact on various domains of brain activity and behavior. In view of the neuronal and endocrine messaging modes of the signaling pathways it is obvious that interoceptive processes take place both at the conscious and subconscious level (Chen et al., 2021). Neuroanatomical and functional imaging studies have provided a detailed mapping of the brain regions in which interoceptive input is received, integrated and distributed to output relays. Whether delivered through neuronal or endocrine pathways, interoceptive information is first processed in subcortical structures of the CNS such as the spinal cord, brainstem and thalamus before it is passed on to higher brain regions including the hypothalamus, insula, anterior cingulate cortex, and somatosensory cortex (Critchley and Harrison, 2013; Wang et al., 2019; Chen et al., 2021). In this way interoceptive signals inform not only regulatory functions of the CNS to maintain internal homeostasis, but also influence feelings (mood, affect, emotion) and their valence as well as motivational and cognitive processes related to preferences, beliefs and decision-making (Figure 1) (Critchley and Harrison, 2013; Wang et al., 2019; Chen et al., 2021). In an experimental setting it has been shown that interoceptive signals from the gut carried by the vagus nerve support memory function in the hippocampus (Suarez et al., 2018).

Particular implications of gut-derived interoceptive signals in mental activity can be deduced from its disturbance in IBS which commonly is associated with visceral hyperalgesia as well as hypersensitivity to emotional challenge. A shown by functional imaging studies, painful rectal distension in healthy controls activates brain regions associated with visceral sensation and interoceptive processing (thalamus, anterior insula, anterior midcingulate cortex), emotional arousal (perigenual anterior cingulate gyrus) and attention and modulation of arousal (inferior parietal, lateral and medial prefrontal cortex) (Tillisch et al., 2011). In IBS patients, the activation of brain regions associated with visceral sensation, interoceptive processing and emotional arousal is significantly increased (Tillisch et al., 2011). In addition, psychological stress in IBS patients enhances the neuronal activation, which rectal distension induces in brain regions such as the insula, midcingulate cortex and ventrolateral prefrontal cortex, to a larger degree than in healthy controls (Elsenbruch et al., 2010). Likewise, IBS patients respond to contextual threat with increased neuronal activity within the salience, attention, default mode and emotional arousal networks within the CNS as compared with healthy controls, which appears to reflect the propensity of IBS subjects to overestimate the likelihood and severity of future abdominal pain (Hong et al., 2016). Furthermore, the hypersensitivity to rectal distension in IBS is related to changes in functional connectivity within resting-state networks associated with interoception, salience and sensory processing, changes that appear to be relevant to the hypervigilance and intestinal hyperalgesia seen in IBS patients (Icenhour et al., 2017). Meta-analyses have shown that patients with IBS present with significantly higher levels of anxiety and depression than healthy controls (Fond et al., 2014). Accordingly, cognitive behavioral therapy and gut-directed hypnotherapy have proved beneficial in a part of IBS patients (Peter et al., 2018; Black et al., 2020b), attesting to gut-brain-gut communication as a viable treatment target.

Background anxiety can strongly influence attitudes, beliefs and decisions, which is most evident in psychiatric disorders associated with generalized emotional disturbances. Decision-making depends on the computation of the value of available options, which in turn are a function of the environment and the internal state of the individual (Paulus and Yu, 2012). Engelmann et al. (2015) have shown that incidental anxiety disrupts the neural valuation of risky decision-making and shifts the valuation focus from possible positive consequences to anticipated negative consequences, a process in which the activity of the anterior insula plays a particular role. Transient anxiety states normally carry adaptive value since they may increase vigilance and attention to possible negative outcomes (Engelmann et al., 2015). This functional anxiety, however, can turn into a maladaptive state if anxious behavior is permanently adopted and becomes detached from the environment (Grupe and Nitschke, 2013). Affect can likewise have an adverse influence on decision-making (Paulus and Yu, 2012; Lerner et al., 2015). For instance, aversive affect appears to be a key source for irrational decision-making, especially with respect to trust in the context of social behavior (Engelmann et al., 2019).

## Interoceptive Gut Signals and “Gut Feelings”: Science Versus Belief

The term “gut feelings” is a popular expression used in everyday language and refers to instinctive feelings, intuition, beliefs and decisions without rational underpinnings (Holzer, 2017). In this context, “gut feelings” are related to positive outcomes as exemplified by notions such as “gut feelings are guardian angels.” The view that feelings originate in the gut may have also been fostered by the labeling of the enteric nervous system as “little brain” or “second brain” (Gershon, 1998). However, feelings and other mental capacities cannot be attributed to this nervous system in the gut, which is indispensable for the neural regulation of digestion (Holzer et al., 2001). Feelings or emotions do not originate in the gut but are generated in the brain, and the term “gut feelings” is a scientifically ill-defined and misleading expression. The impact of gut-derived interoceptive signals and sensations on mental health can be either positive or negative, the latter being aptly exemplified by the neuropsychiatric disturbances in IBS. There is no scientific evidence based on validated data that “gut feelings” have the power to direct judgements and decisions such that they have primarily a beneficial or happy payoff. To the contrary, instantaneous gut sensations known in neurogastroenterology, such as abdominal pain, flatulence, diarrhea-related urgency and nausea, are rather distressful. Notwithstanding these opposing views, the bidirectional communication network between gut and brain and the process of gastrointestinal interoception provide a neurobiological frame to explain emotions, beliefs, judgements and decisions under the influence of signals from the gut (Mayer et al., 2022).

The term “gut feeling” has also entered the scientific literature, which was fuelled not only by the elucidation of the gut-brain communication network but also driven by the entry of the gut microbiota as a factor of the gut-brain axis. Research in experimental models has provided a wealth of information on how the vast microbial community in the gut can participate in gut-brain signaling and interact with the neuronal, immune, endocrine and metabolic messengers of the gut-brain axis (Cryan et al., 2019; Farzi et al., 2019; Gershon and Margolis, 2021; Hassan et al., 2022). However, evidence for a direct impact of the gut microbiota on emotional-affective and cognitive behavior in humans lags behind, and microbiota-directed interventions with proven efficacy in the management of neuropsychiatric disease are not yet available (Dinan and Cryan, 2019; Federici et al., 2020; Simpson et al., 2020; Le Morvan de Sequeira et al., 2022). While changes in the composition and diversity of the gut microbiome are associated with many neurological and psychiatric disorders (Simpson et al., 2021), causal relationships between particular aberrations of the gut microbiome and particular disorders of the human brain remain to be delineated. Despite the insufficient evidence, the hype in microbiome research is also mirrored in the popular press, the vast majority of articles (>90%) reporting health benefits associated with the gut microbiome without mentioning the limitations of such claims (Marcon et al., 2021). “Hope or hype” has become a common phrase in biomedical research areas in which a research boost raises expectations and beliefs in health benefits that await to be fulfilled.

Research hypes also carry the risk of deviating to questionable conceptions. One example relates to the purported mediator of the microbiome-gut-brain axis, 5-hydroxytryptamine (5-HT, serotonin), which both in the scientific and lay press is sometimes said to be an interface between gut microbiota and brain and to act as a “feel-good hormone”. 5-HT synthesized in distinct brain neurons can in fact sustain good mood, and drugs (selective serotonin reuptake inhibitors) targeting the cerebral 5-HT system are efficacious in depression and certain anxiety disorders. However, more than 90% of the body's 5-HT is produced in the gut, primarily in enterochromaffin cells, but also in enteric neurons. Although the gut microbiota can indirectly modify the synthesis of 5-HT in gut and brain through regulating the availability of its precursor L-tryptophan (Gheorghe et al., 2019; Legan et al., 2022), intestinal 5-HT is unlikely to contribute to the “feel-good” action of cerebral 5-HT because it does not pass the blood-brain barrier. To the contrary, an excess of 5-HT in the gut can elicit nausea and emesis associated with chemo- and radiotherapy, facilitate intestinal inflammation, mediate diarrhea associated with bacterial infection, and contribute to IBS-related pain (Gershon, 2013; Legan et al., 2022).

## Conclusion

The gut-brain-gut communication network is part of the interoceptive circuits which enable the brain to sense and interpret the physiological condition in the body and regulate its autonomic and mental activity accordingly. While this relationship has become an important research area in neuroscience, it also provides an example where solid science is at risk going uncritical and fostering unproven conceptions and expectations. It is here that credition research can find fruitful ground to analyze the working of science at the interface of “hype or hope” and to understand how interoceptive signals from the gut impact on mental activity to influence affect, emotion, beliefs, predictions and decisions. In its interdisciplinary approach, credition research is relevant to many areas in which belief processes shape religious, social, societal, economic, legal as well as scientific and medical conceptions and expectations. In analyzing these relationships, credition research bears considerable responsibility to unveil the misinterpretation of scientific data and the neglect of their validity status, which champion unproven notions and predictions. The placebo and nocebo effects represent a particular outcome of belief processes in which a complex set of input information convinces the patient that a certain choice of treatment is better or worse than the other although scientific evidence indicates that they are equivalent in their action. Importantly, placebo and nocebo effects are real, and they work either way, influencing brain activity as indicated by functional imaging studies and altering organ function in the periphery (Meissner, 2014; Bingel et al., 2022).

## Author Contributions

The author confirms being the sole contributor of this work and has approved it for publication.

## Funding

This paper was funded by Dr. Rüdiger Seitz, via the Volkswagen Foundation, Siemens Healthineers, and the Betz Foundation. Siemens Healthineers was not involved in the study design, collection, analysis, interpretation of data, the writing of this article or the decision to submit it for publication.

## Conflict of Interest

The author declares that the research was conducted in the absence of any commercial or financial relationships that could be construed as a potential conflict of interest.

## Publisher's Note

All claims expressed in this article are solely those of the authors and do not necessarily represent those of their affiliated organizations, or those of the publisher, the editors and the reviewers. Any product that may be evaluated in this article, or claim that may be made by its manufacturer, is not guaranteed or endorsed by the publisher.
